# Exposure to source-specific air pollution in residential areas and its association with dementia incidence: a cohort study in Northern Sweden

**DOI:** 10.1038/s41598-024-66166-y

**Published:** 2024-07-05

**Authors:** Anna Oudin, Wasif Raza, Erin Flanagan, David Segersson, Pasi Jalava, Katja M. Kanninen, Topi Rönkkö, Rosalba Giugno, Thomas Sandström, Ala Muala, Jan Topinka, Johan Sommar

**Affiliations:** 1 Department of Public Health and Clinical Medicine, Sustainable Health, Umeå University, Umeå, Sweden; 2 Division of Occupational and Environmental Medicine, Department of Laboratory Medicine, Lund University, Lund, Sweden; 3 Swedish Meteorological and Hydrological Institute, Norrköping, Sweden; 4 Department of Environmental and Biological Sciences, University of Eastern Finland, Kuopio, Finland; 5 A.I. Virtanen Institute for Molecular Sciences, University of Eastern Finland, Kuopio, Finland; 6 Aerosol Physics Laboratory, Physics Unit, Tampere University, Tampere, Finland; 7 Computer Science Department, University of Verona, Verona, Italy; 8 Division of Medicine/Respiratory Medicine, Department of Toxicology and Molecular Epidemiology, Umeå University, Umeå, Sweden; 9 Department of Genetic Toxicology and Epigenetics, Institute of Experimental Medicine of the CAS, Prague, Czech Republic

**Keywords:** Environmental impact, Dementia, Epidemiology

## Abstract

The aim of this study was to investigate the relationship between source-specific ambient particulate air pollution concentrations and the incidence of dementia. The study encompassed 70,057 participants from the Västerbotten intervention program cohort in Northern Sweden with a median age of 40 years at baseline. High-resolution dispersion models were employed to estimate source-specific particulate matter (PM) concentrations, such as PM_10_ and PM_2.5_ from traffic, exhaust, and biomass (mainly wood) burning, at the residential addresses of each participant. Cox regression models, adjusted for potential confounding factors, were used for the assessment. Over 884,847 person-years of follow-up, 409 incident dementia cases, identified through national registers, were observed. The study population’s average exposure to annual mean total PM_10_ and PM_2.5_ lag 1–5 years was 9.50 µg/m^3^ and 5.61 µg/m^3^, respectively. Increased risks were identified for PM_10_-Traffic (35% [95% CI 0–82%]) and PM_2.5_-Exhaust (33% [95% CI − 2 to 79%]) in the second exposure tertile for lag 1–5 years, although no such risks were observed in the third tertile. Interestingly, a negative association was observed between PM_2.5_-Wood burning and the risk of dementia. In summary, this register-based study did not conclusively establish a strong association between air pollution exposure and the incidence of dementia. While some evidence indicated elevated risks for PM_10_-Traffic and PM_2.5_-Exhaust, and conversely, a negative association for PM_2.5_-Wood burning, no clear exposure–response relationships were evident.

## Introduction

The rapid expansion of aging populations presents significant public health challenges, particularly the rising incidence of neurological diseases closely associated with age^1^. Neurological disorders rank among the leading causes of morbidity and mortality globally, constituting 10% of the overall disease burden. Dementia, a broad term encompassing a range of neurological disorders characterized by the progressive decline in memory, language, cognitive abilities, behavior, and problem-solving skills, ultimately impeding daily functioning and social interactions, contributed to approximately 10% of neurological disability-adjusted life years (DALYs) worldwide in 2016^2^. Alzheimer’s disease (AD) and non-Alzheimer dementia (NAD), such as vascular dementia (VaD), stand as some of the most prevalent forms of dementia^3^. Currently, an estimated 160,000 individuals in Sweden live with dementia, equivalent to 1.5% of the entire population. This not only imposes a significant physical and emotional burden on patients and their families but also places substantial strain on the healthcare system and society as a whole^4^.

Given its multifaceted origins, dementia is influenced by a range of genetic, behavioral, and environmental risk factors. With no effective pharmaceutical treatments available, the identification and targeting of modifiable risk factors, specifically those related to behavior and the environment, assume paramount importance. Among the potential environmental factors, air pollution has garnered increasing attention in epidemiological research. In fact, The Lancet Commission on Dementia Prevention, Intervention, and Care has identified air pollution as a significant risk factor for dementia^5^. According to this report, approximately 2% of all dementia cases can be attributed to air pollution. Ambient air pollution constitutes a persistent, long-term exposure that often spans extensive regions, particularly urban areas, affecting a large segment of the population throughout their lifetimes. Consequently, it stands as one of the few environmental risk factors that can be modified at the population level through air quality control measures and mitigation interventions.

Despite a growing body of evidence linking air pollution exposure to dementia, the precise underlying biological mechanisms remain incompletely understood. Proposed pathways include the direct transport of pollutants to the brain through the olfactory bulb, as well as the induction of systemic inflammation and oxidative stress^6^. A pioneering study analyzing MRI data in both children and dogs revealed damage to the prefrontal cortical regions of the brain in individuals exposed to high levels of air pollution^7^. Experimental investigations have also suggested that neuroinflammation and the deposition of ultrafine particles in the brain can lead to enlarged Virchow–Robin spaces, gliosis, and frontal lesions accompanied by vascular pathology in exposed individuals^7–9^. Moreover, animal model studies have provided compelling evidence of the harm associated with air pollution, including impaired blood–brain barrier function, white matter lesions, neuronal degeneration, oxidative damage, glial activation, and neuroinflammation^10,11^. Even short-term exposure to air pollution has been shown to elicit inflammatory responses in brain tissue^12^ and disrupt functional connectivity in the default mode network^13^. In recent years, a substantial body of evidence has thus emerged regarding the role of air pollution in neurodegenerative pathology^14,15^. Notably, an expanding corpus of epidemiological research has explored the impact of fine particulate matter with an aerodynamic diameter of ≤ 2.5 µm (PM_2.5_ on cognitive function and dementia in the elderly population^16–18^. Several studies^19–22^ have highlighted how, for example, PM_2. 5_ is able to cross the blood-alveolar and blood–brain barriers. Because PM_2.5_ is able to promote cellular oxidative stress and systemic inflammatory responses involved in the processes that lead to the onset of respiratory diseases, cardiovascular and neurodegenerative diseases, it represents an important environmental risk factor for public health. It has also been suggested that airborne particulate matter (PM) exposure accelerates brain aging, particularly in ɛ4 carriers, possibly through increased cerebral Aβ production and alterations in hippocampal CA1 neurons and glutamate receptor subunits^23^. In a study with 3029 participants, a 3 µg/m^3^ increase in particles with aerodynamic diameters less than 10 µm (PM_10_) over 10 years was, furthermore, associated with a 1.80% higher baseline level of Aβ1-40 (95% confidence interval (CI) 0.22%, 3.40%), whereas in repeated measures analyses, the association was stronger with a 5.20% increase (95% CI 3.69%, 6.72%)^24^. Similar associations were found for fine particles with an aerodynamic diameter of ≤ 2.5 µm (PM_2.5_) (interquartile range (IQR) 2 µg/m^3^) and nitrogen dioxide (NO_2_) (IQR 7 ppb) in repeated measures analyses^24^.

To establish a causal relationship between long-term air pollution exposure and dementia, well-designed longitudinal observational studies are essential. A comprehensive review focusing on the epidemiological evidence concerning the effects of air pollution on dementia, cognitive function, and cognitive decline among adults conducted an evaluation of eleven prospective cohort studies^25^. Nearly all of these studies reported positive associations between long-term air pollution exposure and the incidence of dementia. However, these studies have notable limitations. For example, many of these studies did not model air pollution concentrations at a spatial resolution high enough to capture local emission variations. Given that local emissions have been associated with higher risk coefficients compared to urban background concentrations^26,27^, it is possible that investigations into urban background air pollution underestimate its health effects. Another shortfall in current evidence lies in the limited assessment of differential associations based on pollutant source. Due to their unique chemical compositions and toxicity, distinct PM sources may exert varying health effects. In one of few studies on source-specific air pollutants and dementia, Shi and colleagues recently found that long-term exposure to PM_2.5_ was significantly associated with higher rates of incident dementia and AD in a large Medicare cohort in the USA and that sulfate, black carbon, and organic matter related to traffic and fossil fuel combustion appeared to be key contributors to these associations^28^. Additionally, there is a lack of studies conducted in regions characterized by relatively low air pollution levels. Such low-exposure settings are also valuable for establishing air quality guideline values.

The primary objective of this study was to evaluate the relationship between total and source-specific concentrations of ambient particulate air pollution, modeled with high spatial resolution, and the incidence of dementia in an area characterized by low levels of air pollution exposure.

## Material and methods

### Study population

The study population comprised individuals enrolled in the Västerbotten Intervention Program (VIP) cohort between January 1, 1990, and December 31, 2014. VIP is a comprehensive program that extends invitations to all residents of Västerbotten county, including those in Umeå municipality, to undergo health assessments upon reaching specific age milestones, including 40, 50, and 60 years (and occasionally 30 years during certain years)^29,30^. These screenings were initiated with the aim of identifying individuals at elevated risk of cardiovascular disease and diabetes through a combination of clinical examinations and interviews covering various risk factors, such as socioeconomic status, education, dietary habits, and physical activity. Prior to their enrollment in VIP, all participants provided informed consent.

To date, over 100,000 individuals have actively participated in the VIP program. Although participation rates have fluctuated between 48 and 67%, they remained consistently high at 66–67% from 1995 to 2005. An analysis conducted in 1998 to assess dropout rates indicated minimal social selection bias. A translated version of the questionnaire is available in the Supplementary Material. For the purpose of this study, residential address histories were acquired from Statistics Sweden records, utilizing the personal identification numbers of each cohort participant. Subsequently, these residential addresses were subjected to geocoding, with an automated process matching them against the Swedish Mapping Cadastral and Land Registration Authority Databases. In cases of inconsistencies or inaccuracies, manual verification and correction of addresses were performed, ensuring the assignment of accurate geographical coordinates. Cohort members from the VIP program were considered for analysis until the time of permanent emigration from the study area, the occurrence of death, or the conclusion of the study period.

### Outcome assessment

We identified cases of dementia using International Classification of Diseases, Tenth Revision (ICD-10) codes F01 and G30. Furthermore, individuals were classified as dementia cases from the commencement of medication treatment (coded as per the Anatomical Therapeutic Chemical code N06D), which was obtained from the Swedish Prescribed Drug Register. Incidence of hospitalization due to dementia was ascertained by cross-referencing participants’ personal identification numbers with the National Patient Register maintained by the Swedish National Board of Health and Welfare (https://www.socialstyrelsen.se/en/statistics-and-data/registers/). Additionally, individuals who neither received a prior dementia diagnosis nor were prescribed dementia medication were included as incident cases if they succumbed to dementia-related mortality. Data on the specific causes of mortality were extracted from the Cause of Death Register of the Swedish National Board of Health and Welfare, again utilizing each participant's personal identification number.

To ensure the exclusion of prevalent cases of dementia, which included those who received a dementia diagnosis within five years before recruitment or were prescribed dementia medication one year before recruitment, we employed strict criteria. Due to the availability of dispensed medication data starting only from 2005 onwards, the follow-up period was initiated in 2006 and concluded in 2015.

### Exposure assessment

**Figure 1:**
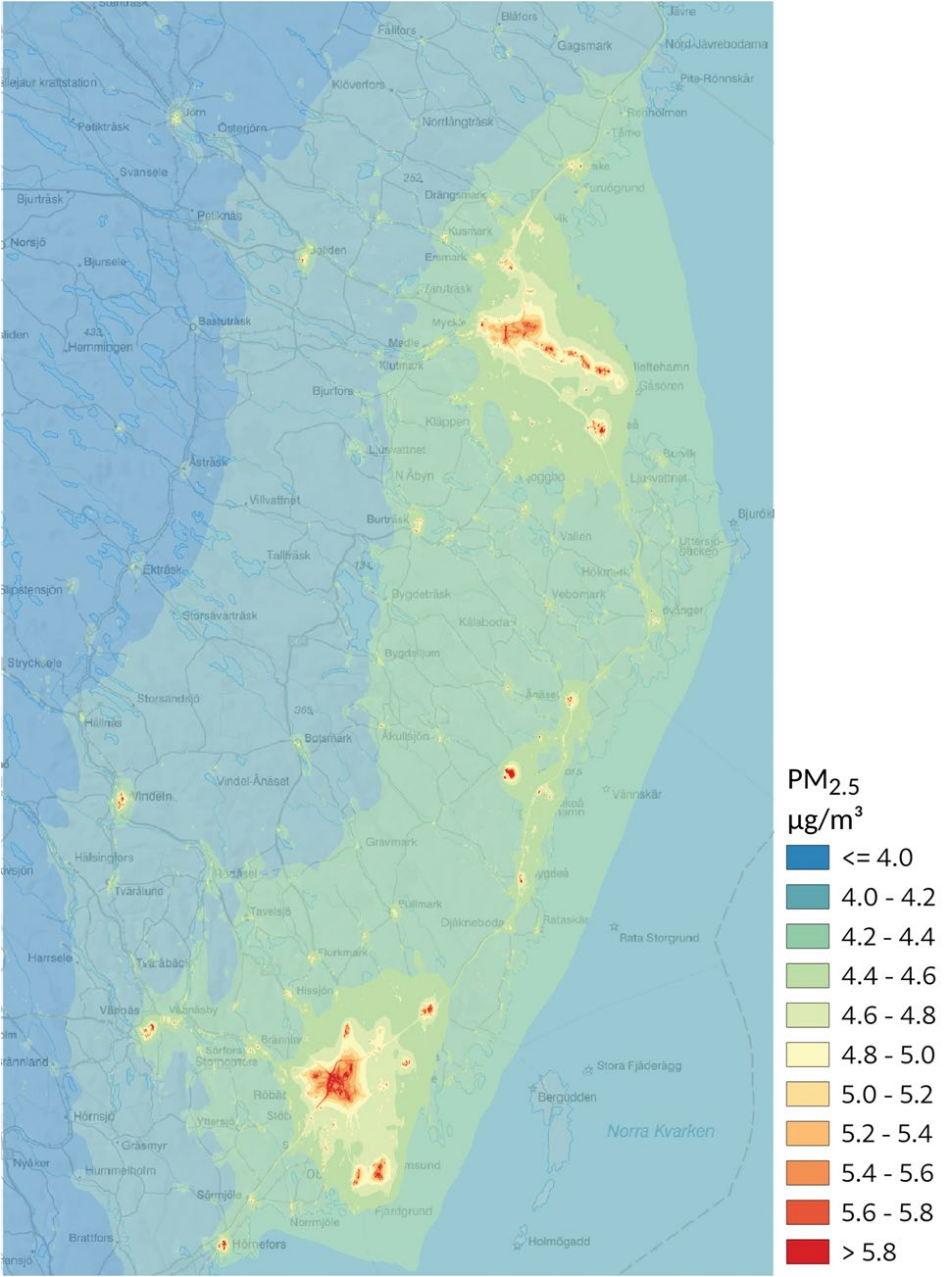
Modelled concentrations of total PM_2.5_ during 2011 in the study area.

The Västerbotten county, situated in Northern Sweden, experiences notably low air pollution levels when viewed from an international perspective, particularly in terms of contributions from long-range sources (Fig. 1).

A comprehensive description of the dispersion model employed for exposure assessment has been previously documented^31^. In brief, emission inventories for the years 2000 and 2011 served as the foundation for Gaussian dispersion model simulations of particulate matter concentrations, specifically targeting PM_10_ and PM_2.5_. While the modeling occurred on an hourly basis, the results were consolidated into yearly average concentrations for our analyses. To obtain annual average concentrations for years falling between the modeled years (2000 and 2011), linear interpolation was utilized, assuming gradual emission changes over the intervening years. These interpolated values were also adjusted by a meteorological index, derived from continuous dispersion simulations conducted over a limited geographical area throughout the entire period (2000–2011). This adjustment accounted for year-to-year meteorological variations. For 2012 and 2013, concentrations were extrapolated following the same approach, assuming no significant alterations in emissions since 2011. To address pronounced spatial concentration variations near roads and in proximity to chimneys associated with residential stoves and boilers, a quadtree receptor grid was employed, achieving a spatial resolution as fine as 35 m × 35 m. For inner-city streets flanked by buildings on one or both sides, an additional concentration component was simulated using the Operational Street Pollution Model (OSPM)^32^. PM-exhaust attributable to road traffic, which considers different vehicle types, speeds, and driving conditions, was computed based on the Handbook on Emission Factors for Road Traffic, version 3.1^33^. Non-exhaust emissions stemming from traffic-related PM primarily encompass particles from road wear, with a minor contribution from brake and tire wear^34–36^. The contribution of non-exhaust emissions to the yearly mean concentrations was estimated under the assumption of a spatial distribution identical to that of exhaust emissions. The exhaust-to-non-exhaust emissions ratio was estimated for each modeling year using methodologies detailed in Omstedt et al.^36^. Emissions for small-scale residential heating (PM-wood burning) were derived from a comprehensive inventory encompassing individual privately owned stoves and boilers, supplemented with data from chimney sweepers and insights into wood-burning habits obtained through interviews^31^. Industrial and energy production facilities were represented as point sources within the model. Emissions from shipping were also incorporated, with a methodology akin to that described by Jalkanen et al.^35^.

Annual mean concentrations of long-range transported PM_10_ and PM_2.5_ were predicated on measurements from regional background stations. Given the demonstrated small-scale spatial variation between regional background sites^37^, no additional adjustments were applied within the assessment area. Total annual concentrations of PM were computed as the sum of these regional background measurements and locally modeled concentrations. In a previous study, the validation of modelled concentrations against measurements from an urban background site in the study area yielded a 17% average difference across six different years for PM_2.5_, whereas the corresponding number was 49% for measurements taken at a traffic site in the study area^31^. For PM_10_, the corresponding average differences were 10% for the urban background site and 2% for the traffic site^31^.

Ultimately, the resultant concentrations encompassed total PM_10_, total PM_2.5_, and local, source-specific PM concentrations (PM_10_-Traffic, PM_2.5_-Exhaust, PM_2.5_-Wood burning) were estimated for each study participant's residential address. Changes in address during the exposure assessment period (2000–2013) were duly accounted for. PM_10_-Traffic encompassed particles originating from exhaust sources (primarily PM_2.5_) as well as non-exhaust particles stemming from road wear, brake, and tire wear (primarily PM_2.5–10_).

### Confounders

To address potential confounding factors, various covariate data were gathered, including calendar year, gender, smoking status (current, former, never smoker), alcohol consumption (daily, weekly, seldom, never), physical activity during leisure time (sedentary, moderate, intermediate, or vigorous), marital status (single, married or cohabiting, data missing), educational attainment (primary school or lower, up to secondary school or equivalent, university degree or higher, data missing), and employment status (employed, unemployed/not gainfully employed, retired, data missing). Furthermore, the socioeconomic status at the area level was estimated based on each cohort member's mean neighborhood income. This calculation utilized individual incomes of individuals of working age for the calendar year 1994, as reported by Small Areas for Market Statistics (SAMS).

### Statistical methods

We employed Cox-proportional hazard models to estimate hazard ratios (HR) for dementia in association with various PM sources (total PM_10_, total PM_2.5_, PM_10_-Traffic, PM_2.5_-Exhaust, and PM_2.5_-Wood burning). Age served as the underlying time variable for the baseline hazard. The regression model was adjusted for calendar year, baseline individual risk factors, and area-level socioeconomic status, with consistent covariate inclusion across all analyses. PM associations were evaluated using two exposure windows: a moving average over 1–5 years before the event (lag 1–5) and 6–10 years before the event (lag 6–10). To be included, annual mean air pollution concentrations were required to be available for at least 80% of the time window. Both single- and two-pollutant models were employed. HRs and their corresponding 95% CIs were presented in tertiles of PM. Due to low statistical power, it was not possible to evaluate concentration-responses at a great many points, hence the choice of tertiles for exposure rather than, for example, quartiles or cubic splines.

### Ethical approval

The study underwent review and received approval from the Ethical Review Board in Umeå (reference 2015/16-31Ö). All methods were carried out in accordance with relevant guidelines and regulations.

## Results

### Participant characteristics

The study encompassed 70,057 individuals, with 53% being women, and a median age of 40 years at recruitment. Regarding individual risk factors, 20% were current smokers, 28% were former smokers, and 57% consumed alcohol on a weekly basis (Table 1). Furthermore, 40% did not engage in exercise that required training attire during their leisure time. In terms of socioeconomic status, 21% of cohort members were not in a domestic partnership, 38% had an education level of primary school or lower, and 6% were unemployed. The distribution of these individual risk factors is presented in Table 1, further stratified by estimated PM concentrations, using PM_10_-Traffic as an illustration. In the highest tertile of PM_10_-Traffic, there was a slightly higher proportion of female participants, individuals who had never smoked, those who engaged in regular physical activity during leisure time, individuals with higher education levels, and gainfully employed individuals. Over a total of 884,847 person-years of follow-up, we identified 409 incident cases of dementia.

**Table 1:** Descriptive statistics of study participants including individual risk factors, area level socioeconomic status and stratification by tertiles of PM_10_-traffic.

	All	Tertiles of PM_10_-Traffic		
		< 0.21 µg/m^3^	0.21–0.61 µg/m^3^	> 0.61 µg/m^3^
Participants (n)	70,057	23,353	23,352	23,352
Sex				
Women	53%	50%	53%	54%
Smoking status				
Current smoker	20%	19%	21%	20%
Former smoker	28%	28%	28%	30%
Never smoker	45%	45%	45%	45%
Missing	7%	9%	6%	6%
Alcohol consumption				
Weekly	56%	47%	56%	55%
Seldom	43%	41%	42%	44%
Never	1%	1%	1%	1%
Missing	1%	1%	1%	1%
Leisure time physical activity				
Sedentary	36%	36%	35%	35%
Moderate	41%	40%	42%	43%
Intermediate or vigorous	21%	22%	22%	20%
Missing	2%	2%	2%	1%
Marital status				
Married or living with a partner	78%	80%	76%	77%
Education level				
Primary school or lower	32%	32%	32%	32%
Up to secondary school or equivalent	27%	30%	28%	24%
University degree or higher	34%	30%	34%	39%
Missing	7%	8%	6%	5%
Employment status				
Gainfully employed	81%	79%	81%	83%
Unemployed/not gainfully employed	5%	5%	5%	5%
Retired	4%	4%	4%	3%
Missing	10%	11%	9%	9%
Mean neighbourhood income (SEK)	121,669	113,270	124,549	127,226

BMI, body mass index, measured as weight (kg)/height (m)^2^; SD, standard deviation.

### Particle concentrations

Mean lag 1–5 concentrations of total PM_10_, total PM_2.5_, PM_10_-Traffic, PM_2.5_-Exhaust and PM_2.5_-Wood burning were 9.50, 5.61, 0.51, 0.10 and 0.78 µg/m^3^ during follow-up (Table 2). Overall, lag 6–10 concentrations were similar to lag 1–5 concentrations but lag 6–10 concentrations for total PM_2.5_ were slightly higher due to a decreasing trend in total PM concentrations during the study period. Total PM_2.5_ concentrations were correlated with PM_10_-Traffic and PM_2.5_-Wood burning (r = 0.3 and r = 0.4, respectively, for lag 1–5); however, the correlation between PM_10_-Traffic and PM_2.5_-Wood burning was low (r = 0.06 for lag 1–5).

**Table 2:** Air pollution concentrations for the person-years of follow-up presented as means and standard deviations (SD) together with quartile limits and inter-quartile ranges (IQRs).

	Mean (SD)	Median (1st quartile–3rd quartile)	IQR
Lag 1–5			
Total PM_10_	9.50 (1.38)	9.19 (8.58–10.24)	1.67
Total PM_2.5_	5.61 (0.81)	5.46 (5.08–6.05)	0.97
PM_10_-Traffic	0.51 (0.61)	0.34 (0.21–0.61)	0.40
PM_2.5_-Exhaust	0.10 (0.12)	0.08 (0.04–0.12)	0.08
PM_2.5_-Wood burning	0.78 (0.33)	0.75 (0.61–0.92)	0.32
Lag 6–10			
Total PM_10_	9.76 (1.39)	9.48 (8.79–10.53)	1.75
Total PM_2.5_	5.75 (0.81)	5.61 (5.20–6.23)	1.03
PM_10_-Traffic	0.49 (0.60)	0.33 (0.20–0.59)	0.39
PM_2.5_-Exhaust	0.11 (0.13)	0.08 (0.05–0.12)	0.08
PM_2.5_-Wood burning	0.76 (0.32)	0.73 (0.59–0.89)	0.30

### Dementia incidence

#### Single-pollutant models

**Figure 2:**
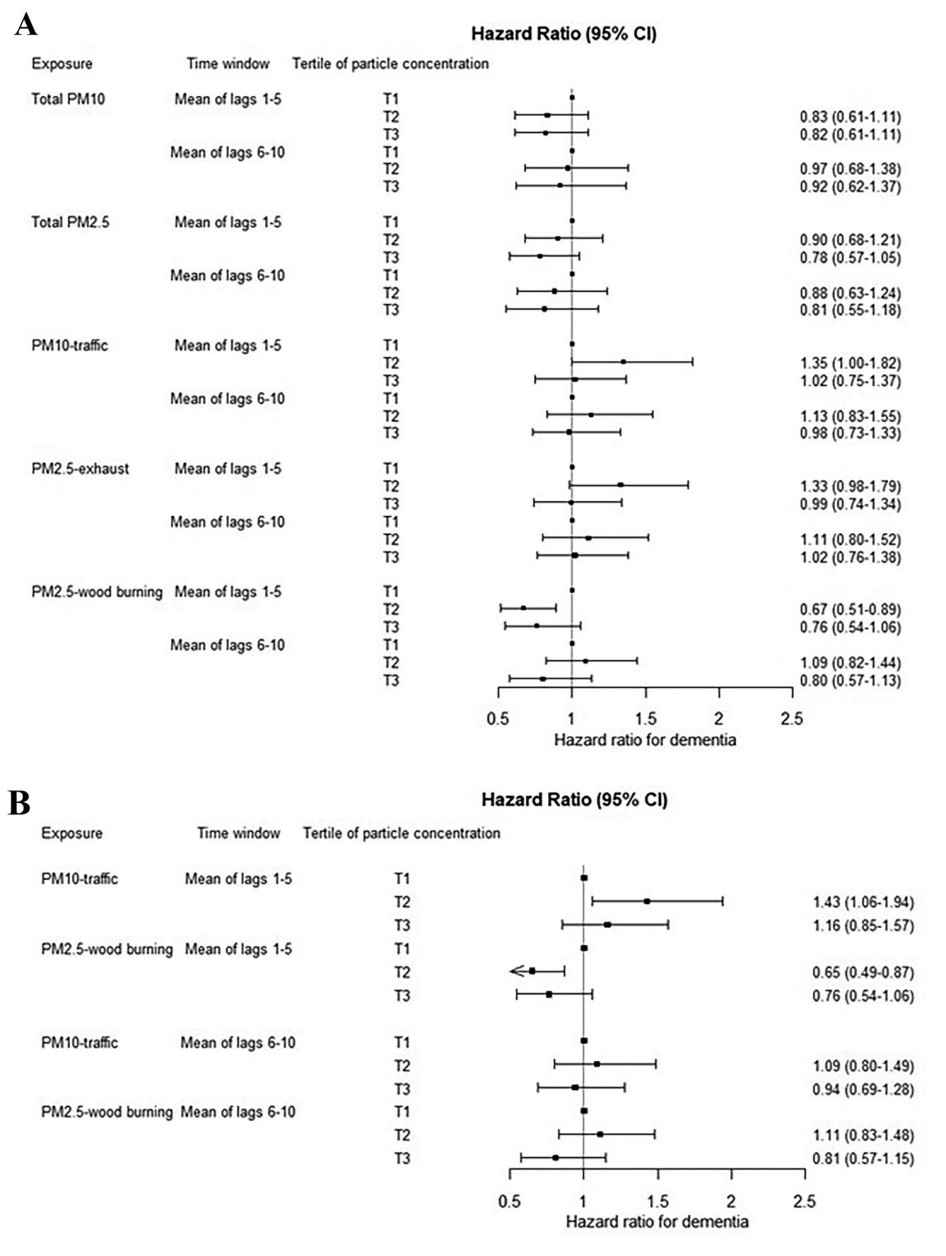
(**A**) Associations between tertiles of particulate matter (PM) air pollution and dementia in single-pollutant models. (**B**) Associations between tertiles of particulate matter (PM) air pollution and dementia in a two-pollutant model including both PM_10_-Traffic and PM_2.5_-Wood burning.

For concentrations of total PM_10_ for lag 1–5, the risk estimates for dementia incidence were 17% (95% CI −39 to 11%) and 18% (95% CI − 39 to 11%) lower in the second and third exposure tertiles, respectively, compared to the first (Fig. 2A). These risk reductions were lower compared to lag 6–10 for total PM_10_. For analyses on total PM_2.5_, decreased risks were also observed. Estimates for both total PM_10_ and total PM_2.5_ were low in precision.

Concerning PM_2.5_-Exhaust, the risk estimates for incident dementia were 33% (95% CI − 2 to 79%) and 11% (− 20 to 52%) higher in the second exposure tertile compared to the first for lag 1–5 and lag 6–10, respectively (Fig. 2A). No risk increases were observed in the third tertile, however. Risk estimates for PM_10_-Traffic were very similar to those for PM_2.5_-Exhaust. Regarding PM_2.5_-Wood burning, risk estimates for lag 1–5 were 33% (95% CI − 49 to − 11%) and 24% (95% CI − 46 to 6%) lower in the second and third exposure tertiles, respectively, compared to the first. For lag 6–10 of PM_2.5_-Wood burning, the risk estimate was 9% (95% CI − 18 to 44%) higher in the second tertile but 20% (95% CI − 43 to 13%) lower in the third tertile compared to the first.

#### Two-pollutant model

In the two-pollutant model including both lag 1–5 PM_10_-Traffic and PM_2.5_-Wood burning, the risk of incident dementia was 43% (95% CI 9–94%) higher in the second exposure tertile but only 16% (95% CI − 15 to 57%) higher in the third tertile compared to the first (Fig. 2B). In relation to lag 6–10 exposure, the single-pollutant model estimates did not significantly change following the simultaneous adjustment of PM_10_-Traffic and PM_2.5_-Wood burning.

## Discussion

### Main findings

While there were indications of a potential association between dementia risk and particulate matter from traffic sources (PM_10_-Traffic and PM_2.5_-Exhaust), it appeared that the relationship was not strictly linear. Conversely, concerning total PM_10_, total PM_2.5_, and PM_2.5_-Wood burning, there were hints of reduced dementia risk in the higher exposure tertiles, albeit with limited precision. Conducting research in low exposure settings is crucial for establishing air quality guideline values, making this study a significant addition to our existing knowledge despite the somewhat inconsistent findings.

### Evidence on total particulate matter and dementia incidence

While The Lancet Commission on Dementia Prevention, Intervention, and Care report indicated that 2% of dementia cases could be attributed to air pollution^5^, the evidence regarding the association between dementia incidence and total PM_2.5_ exposure has yielded mixed results. For instance, a study using the Women's Health Initiative Memory Study (WHIMS) cohort found no significant link between annual PM_2.5_ exposure (IQR 3.9 µg/m^3^) and dementia incidence^38^, consistent with the findings of our present study. The Rotterdam study also failed to detect a clear association between air pollution exposure (modelled levels of PM_10_, PM_2.5_, and NO_2_) and the risk of dementia or cognitive decline^39^. However, a longitudinal, population-based study employing data from the Swedish National Study on Aging and Care in Kungsholmen demonstrated a 54% increased risk of dementia per inter-quartile range difference (IQR 0.88 µg/m^3^) of PM_2.5_, although most of this association could be attributed to cardiovascular comorbidities^27^. Another study involving seven European cohorts in low exposure settings found no association between air pollution and dementia mortality^40^.

On the other hand, most studies have reported positive associations. A recent meta-analysis, where two new cohorts in China were included, revealed that dementia risk increased with exposure to PM_2.5_, PM_10_, NO_2_, and nitrogen oxides (NO_X_)^41^. In the Betula study, which was conducted partly in the same study area as our present study, we observed that each 1 µg/m^3^ difference in annual mean PM_2.5_ concentration was associated with a hazard ratio of 1.23 (95% CI 1.01–1.50) for dementia^42^. The Rome Longitudinal Study, utilizing a large administrative cohort, reported mixed results, with exposure to NO_X_, NO_2_, PM_2.5_, and PM_10_ showing negative associations with AD but positive associations with vascular dementia^43^. Another study reported a positive association between PM_2.5_ exposure and dementia incidence, albeit not statistically significant^44^. In contrast, a different study within the same cohort reported contrasting results and found a 92% increased risk of dementia in the fourth quartile of PM_2.5_ exposure compared to the first^23^. A Canadian study discovered a positive association, citing a 4% increased risk for every IQR (3.4 µg/m^3^) increase in PM_2.5_ exposure^45^. In a London-based study, a 6% increased incidence of all-cause dementia was identified for each IQR (0.95 µg/m^3^) increase in PM_2.5_ exposure^26^. Additionally, an IQR increase in baseline PM_2.5_ exposure was associated with a 9% increase in dementia mortality in the United States Veterans Health Administration (VA) study^46^. A recent analysis within the Three-City Study (3C Study) showed a 20% increased risk for incident dementia per 5 µg/m^3^ increase in PM_2.5_ concentrations modelled using land-use regression^47^. Using a prediction model based on machine learning algorithms, another study reported a 6% increase in incident dementia per IQR increase in the 5 years average PM_2.5_^48^.

In contrast to these studies, the present study did not find a positive association between total PM_10_ or total PM_2.5_ and incident dementia. There are several potential explanations for this difference. The study area has exceptionally low levels of total PM_2.5_, which might indicate that air pollution at such low levels does not contribute significantly to dementia. Roughly 60–70% of the urban background concentrations contributing to total PM_10_ and total PM_2.5_ consist of secondary, long-range transported (LRT) PM, which has been associated with lower risk coefficients compared to locally-produced, near-source air pollution. Recent studies on the association between air pollution and all-cause mortality have suggested that effect estimates may be considerably higher for locally produced air pollution, also known as “near-source” pollution, compared to regional background air pollution^49^. Consequently, LRT PM is less likely to drive health impacts typically associated with local sources, such as road traffic emissions and residential wood burning. The lack of association for total PM_10_ and total PM_2.5_ could also be attributed to the presence of residential wood burning within these exposure variables. Indeed, residential wood burning is considered a significant component of urban background concentrations in the study area^50^ and was found to be negatively associated with dementia.

Many of the previous studies have explored linear associations with PM as a continuous variable. However, in our present study, linear associations between dementia incidence and total PM_10_ or PM_2.5_ were not analyzed, as the tertile analyses did not suggest any exposure–response relationships. Quartiles and cubic splines were initially explored to thoroughly investigate potential non-linear associations. However, the statistical power was insufficient for these analyses.

### Evidence on source-specific particulate matter and dementia incidence

#### Road traffic-related sources

While few studies on the relationship between air pollution exposure and dementia have explored emissions from local sources, the findings of our present study are largely consistent with existing research investigating PM from traffic and exhaust sources. For example, a study conducted in England, which generally experiences higher levels of air pollution, reported an 8% increased risk of dementia per 0.58 µg/m^3^ change (IQR) in dispersion modelled PM_2.5_ from traffic^26^. Similarly, a Swedish study that analysed the Betula cohort, characterized by air pollution concentrations like our study, found a 66% increased risk of incident dementia for individuals in the third quartile of PM_2.5_ exposure from traffic exhaust (0.14–0.24 µg/m^3^) compared to the first quartile (0.017–0.086 µg/m^3^)^51^. The magnitude of the association for fourth quartile exposure (0.24–1.81 µg/m^3^) in the Betula study (HR 1.41, 95% CI 0.97–2.04) closely resembles our findings. In our study, the observed elevated risk of dementia for participants exposed to PM_10_-Traffic concentrations in the second tertile (0.21–0.61 µg/m^3^) was 43% compared to the first tertile (< 0.21 µg/m^3^) in the two-pollutant model. Since the estimate for the third tertile was lower (an increase of 16% compared to the first tertile) than the second in the two-pollutant model, we refrained from assuming linearity when analyzing the associations for PM from traffic and exhaust sources.

Beyond directly assessing PM from various traffic-related sources, numerous studies examining dementia incidence have employed proxy measures for traffic-related air pollution, such as modelled NO_X_ and NO_2_, most of which have reported positive associations. In the Betula study, for example, individuals exposed to the highest quartile of NO_X_ (> 26.0 µg/m^3^) had a 60% increased risk of incident dementia compared to those in the lowest quartile (4.8–9.0 µg/m^3^)^52^. A follow-up analysis of the Betula study also found a 48% increased risk of dementia for individuals in the third quartile of NO_X_ exposure (> 17.0–26.0 µg/m^3^) compared to the first quartile (4.8–9.0 µg/m^3^)^53^. When the analysis was restricted to APOE ε4-positive Betula cohort participants , the study revealed that those in the third (> 17.0–26.0 µg/m^3^) and fourth (> 26.0 µg/m^3^) quartiles of NO_X_ exposure had a 59% and 48% elevated risk, respectively, for incident dementia compared to the first quartile (4.8–9.0 µg/m^3^)^54^. Another study in the Betula cohort suggested that the associations between PM_2.5_ and dementia were more pronounced among APOE ε4-positive participants and those with below-average odor identification ability^34,42^. However, there is some heterogeneity in the literature, as a recent large, pooled European cohort study found no association between NO_2_ exposure and dementia mortality^40^. This variation may arise from NO_2_ primarily being a secondary pollutant and, therefore, having different correlations with traffic exhaust particles. In a Canadian cohort study, which employed residential proximity to traffic (> 50 m) as a proxy for traffic-related air pollution, a 7% increased risk of incident dementia was observed^45^.

In our present study, PM was chosen as the focus because the adverse effects of air pollution on the brain are primarily attributable to PM^55^. Moreover, understanding whether specific air pollution sources have distinct health effects is critical when designing strategies for air pollution mitigation.

#### Residential wood (biomass) burning

On the contrary, our findings regarding PM-wood burning do not align with previous research. For example, the Betula study reported a 55% increased risk of incident dementia per 1 µg/m^3^ of modelled PM_2.5_ from local residential wood burning. This association was even more pronounced among individuals with wood-burning stoves in their homes compared to those without^51^. Surprisingly, despite sharing a substantial portion of the same study area, our present study observed a negative association for PM-wood burning. Several potential explanations could account for this unexpected outcome. One possibility is the presence of residual confounding due to urban and rural socioeconomic gradients, including differences in education^56^. As extensively documented in the literature, lower education levels, which are typically more prevalent in rural areas than in urban ones, constitute a significant social determinant of health^56–59^. Additionally, lower education levels have been linked to an increased risk of dementia^60^. Paradoxically, individuals with higher socioeconomic status (SES) may be more inclined to use hospital outpatient consultations^60,61^. Consequently, higher SES groups might be more likely to receive dementia diagnoses and prescriptions for dementia medication, potentially leading to differential misclassification. Furthermore, reports have indicated an inverse relationship between ambient PM and socioeconomic status^62,63^. If individuals with higher SES are, on average, exposed to lower air pollution concentrations, this negative bias could explain the protective effect estimates observed for PM-wood burning in our study.

In addition to this possibility, the lack of association for PM-wood burning might also be attributed to its composition. Specifically, PM emissions from traffic, particularly PM-exhaust emissions, more commonly consist of ultrafine particles^64^ than PM from wood burning^65^. While ultrafine particles emitted from traffic do not significantly contribute to the overall particulate mass from traffic sources (PM_2.5_-Exhaust and PM_10_-Traffic in our study), they are highly correlated with them^64^. Generally, smaller particle sizes are associated with more efficient deposition of particles into the human body, allowing them to cross the blood–brain barrier and potentially impact brain health. Consequently, the unique composition of PM from a specific source can influence its potential to induce adverse health outcomes.

### Methodological considerations

Our study boasts several noteworthy strengths, including its prospective cohort design, large population size, extensive follow-up period, and high-quality data on clinically diagnosed dementia cases. In contrast, many prior studies have primarily relied on register-based data or hospital discharge records, which frequently fail to comprehensively capture dementia cases, resulting in an underestimation of the true number of cases. Indeed, a Swedish data study revealed that while specificity was high when using register data to ascertain dementia diagnoses, sensitivity was lower, leading to the oversight of nearly half of all dementia cases^66^. Although our present study incorporated data from three well-validated sources (National Patient Register, Swedish Cause of Death Register, and Swedish Prescribed Drug Register), it remains possible that some dementia cases were not identified.

Another notable strength is our assessment of air pollution exposure at each individual's residential address using high-resolution spatio-temporal dispersion modeling. These modelled concentrations were validated against air pollution measurements and exhibited strong agreement with most monitoring stations. Furthermore, we accounted for changes in residential addresses during the follow-up period using data from the Swedish Population Register. Our ability to adjust for potential confounding factors also represents a critical strength.

However, there are limitations to consider related to potential exposure misclassification. For instance, the accuracy of the air pollution models is a source of exposure misclassification. Validation of the model indicated that the differences between modelled and measured levels may be substantial, especially for PM_2.5_, which could have caused bias, most likely towards the null. Additionally, assessing air pollution exposure solely at residential addresses may not accurately reflect personal exposure, which includes exposure at workplaces, during commutes, and indoor environments at home. While non-differential, this exposure misclassification can reduce precision. Differential misclassification, however, can introduce bias in various directions, both toward and away from the null, and can occur with respect to both the outcome and the exposure. In our study, air pollution concentrations slightly increased with higher individual education levels, which are strongly associated with dementia risk, and neighborhood-level income (Table 1). If outcome misclassification also depended on socioeconomic status, it could suggest a risk of differential misclassification. However, assessing whether such misclassification is a plausible explanation for the lack of findings in our study is challenging. While the associations were adjusted for several important confounders, residual confounding or effect modification may still be present, as we lacked data on other environmental risk factors such as noise exposure and proximity to green spaces near residential addresses. Nonetheless, a study on air pollution and dementia in an overlapping study area found that adjusting for noise did not substantially influence effect estimates^67^. Additionally, we lacked information on occupational factors, which may be related to dementia risk^68^. Exploring factors like shift work would have been particularly interesting^69,70^.

Our study’s exposure window was limited to the last ten years, and we could not investigate the potential role of earlier-life exposure. Finally, our findings may not be generalizable to settings with high concentrations of air pollution since our study was conducted in an area where, from a global perspective, total air pollution concentrations are relatively low. Nevertheless, concentrations of locally produced air pollution in this study area are quite similar to those in other cities in Sweden and other Nordic countries, which may support the generalizability of our results to such settings.

### Future research

Further research on the association between air pollution, particularly PM and its locally produced sources^71^, and dementia is required, as current evidence on this environmental risk factor is somewhat inconsistent. Such studies are especially lacking in low- and middle-income countries where air pollution concentrations, sources and, thereby, its composition, can differ substantially from that of high-income countries. To date, there are furthermore very few studies on short-term exposure to air pollution and outcomes related to brain function, although short-term pollution-attributable decrements in default mode network functional connectivity was recently observed in a human exposure study^12^. Life course air pollution exposure in relation to cognitive health is another under-studied area, where more studies are needed^72^.

In this unique low-exposure setting located in Northern Sweden, our present study contributes valuable insights to the ongoing discourse regarding the potential link between long-term exposure to local sources of particulate air pollution and the risk of dementia incidence. Our findings, while shedding light on this complex issue, do not provide definitive evidence of a clear linear association in this specific context.

The significance of our study lies in its meticulous examination of a region characterized by relatively low levels of air pollution, especially when viewed from an international perspective. This distinct setting allows us to explore the impact of air pollution in an environment where total air pollution concentrations are notably lower than those observed in many other parts of the world.

However, despite the rigorous methodology employed and the wealth of data collected, our results do not unequivocally establish a direct link between long-term exposure to local sources of particulate air pollution and an increased risk of developing dementia. While some previous research has suggested such associations, our study, conducted in an area with low air pollution levels, does not provide robust confirmation of these claims. Future studies should, furthermore, aim to investigate potential non-linearities of such associations, which would require larger statistical power than in the present study. Indeed, some findings indicate that exposure to outdoor air pollutants, particularly PM_10_, may non-linearly increase the risk of mild cognitive impairment progressing to dementia once a certain ambient air concentration threshold is surpassed^73^.

It's important to recognize that the relationship between air pollution and dementia is intricate and multifaceted. Our study adds to the growing body of literature on this topic but underscores the need for further investigation in diverse geographic settings and with various levels of air pollution exposure. The absence of a definitive link in this low-exposure context highlights the complexity of this research area and the potential influence of local factors on the observed outcomes.

## Conclusion

In conclusion, our study underscores the importance of considering the specific environmental context when assessing the impact of air pollution on health outcomes. While it does not provide conclusive evidence of a direct connection between local sources of particulate air pollution and dementia incidence in this low-exposure setting, it contributes valuable data to the broader scientific understanding of this critical issue. Future research in different environments will be instrumental in unraveling the complex relationship between air quality and cognitive disorders.

### Supplementary Information

Supplementary Information.

## Data Availability

The data that support the findings of this study are available from Region Västerbotten but restrictions apply to the availability of these data, which were used under license for the current study, and so are not publicly available. Data are however available from the authors upon reasonable request and with permission of Region Västerbotten.
