# Supplementary material for: Exposure to source-specific air pollution in residential areas and its association with dementia incidence: a cohort study in Northern Sweden
Source: Sci Rep. 2024 Jul 5;14:15521. doi: 10.1038/s41598-024-66166-y (PMC11226641; doi:10.1038/s41598-024-66166-y)
Supplement: Supplementary file 1 — Supplementary Information. [file 41598_2024_66166_MOESM1_ESM.pdf]

VÄSTERBOTTEN INTERVENTION PROGRAM
HEALTH SURVEY

This translated version of the VIP questionnaire is to facilitate filling in the Swedish Questionnaire. Use it as a help to answer the Swedish questionnaire

This version cannot be optically scanned and therefore your answers here cannot be used in the medical case report.

Thank you

SECTION A:

Name……………………………………………………………………………………

Personal ID number YY/MM/DD-XXXX…………………………………

SECTION B: SOCIO-ECONOMIC STATUS

B1. What is your marital status?

1. Single
2. Married / Living with spouse
3. Widow / Widower
4. Divorced / Separated
5. Remarried

B2. What is the highest level of education you have completed?

1. Elementary school
2. Comprehensive school
3. Residential college for adult education
4. Senior High School
5. ”Girls school”
6. Vocational training school
7. Upper secondary School
8. High-school
9. College/university

B3. Whom are you presently living with?

1. Adult (husband, wife, spouse)
2. Child
3. Sibling
4. Mother
5. Father
6. Both parents
7. Other
8. I live alone

B4. What’s your profession?
(State your main occupation/profession. If you do not work currently, please state your previous occupation/profession)

Profession:____

Do you work shifts / weekends?
1. Yes
2. No

Have you been on sick-leave for more than six months?
1. Yes
2. No

B5. What is your present type of employment/position?

1. Permanently employed
2. Temporary post
3. Working in the home
4. Unemployed

5. Student
6. Elf-employed/run own business
7. Early retirement pension, full
8. Early retirement pension, partial

B6. What country are you from?

1. Sweden
2. Other ________________

SECTION C: HEALTH STATUS

C1. How do you rate your own health during the past year?

1. Very good
2. Pretty good
3. Somewhat good
4. Pretty bad
5. Bad

C2. Did any of your parents or siblings suffer a heart attack/myocardial infarction or stroke before age 60 years?

1. Yes
2. No
3. Don't know

C3. Does any of your parents or siblings suffer from diabetes?

1. Yes
2. No
3. Don't know

C4. Have you at any time been informed by a doctor or other medical personnel that your blood pressure is high?

1. Yes
2. No

C6. Do you suffer from diabetes?

1. Yes
2. No

C7. If you have answered "Yes" on question C6, what is your treatment?

1. Diet and exercise
2. Tablets
3. Insulin
4. None of the above

C8. Have you been hospitalized for a verified heart attack/myocardial infarction?

1. Yes → What year?
2. No

C9. *To be answered of women only*
Have you had gestational diabetes?

1. Yes
2. No

C10. Have you in the past two weeks used any of the following medicines? (Mark suitable alternative)

1. Yes, medication for hypertension
2. Yes, medication for angina pectoris/ medicine for the heart
3. Yes, sedative or sleep agent
4. Yes, medication for gastric ulcer/gastritis
5. Yes, lipid lowering drug
6. Yes, painkiller
7. No, I do not use any of the above medicines

C11. Did you during the past two weeks take any other medications that was prescribed by a doctor (for example for depression, for epilepsy, penicillin, hormones) or any medication/drug that was not prescribed by a doctor (for example, aspirin, vitamins, iron, omega-3 or other dietary supplements, nature cures or other)? Also include items that you tick on question L2.
1. Yes
2. No

C12. If you answered "yes" to any of the alternatives in question C10 and C11, please state the exact name of all medications/drugs/supplements, the kind of preparation (for example tablet, spray, suppository, vaginal insert, salve), the dose, and if you take this on a daily basis or when needed.)? Also include items that you tick on question L2.

Name of medication	Kind of preparation	Dose	Daily	When needed

RAND 36-Item Health Survey 1.0 Questionnaire Items

(This questionnaire was developed at RAND® as part of the Medical Outcomes Study. RAND® corporation)

1. In general, would you say your health is:	
Excellent	1
Very good	2
Good	3
Fair	4
Poor	5

2. **Compared to one year ago**, how would your rate your health in general **now**?	
Much better now than one year ago	1
Somewhat better now than one year ago	2
About the same	3
Somewhat worse now than one year ago	4
Much worse now than one year ago	5

The following items are about activities you might do during a typical day. Does **your health now limit you** in these activities? If so, how much?

(Circle One Number on Each Line)

	Yes, Limited a Lot	Yes, Limited a Little	No, Not limited at All
3. **Vigorous activities**, such as running, lifting heavy objects, participating in strenuous sports	[1]	[2]	[3]
4. **Moderate activities**, such as moving a table, pushing a vacuum cleaner, bowling, or playing golf	[1]	[2]	[3]
5. Lifting or carrying groceries	[1]	[2]	[3]
6. Climbing **several** flights of stairs	[1]	[2]	[3]
7. Climbing **one** flight of stairs	[1]	[2]	[3]
8. Bending, kneeling, or stooping	[1]	[2]	[3]

9. Walking **more than a mile**	[1]	[2]	[3]
10. Walking **several blocks**	[1]	[2]	[3]
11. Walking **one block**	[1]	[2]	[3]
12. Bathing or dressing yourself	[1]	[2]	[3]

During the **past 4 weeks**, have you had any of the following problems with your work or other regular daily activities **as a result of your physical health**?

(Circle One Number on Each Line)

	Yes	No
13. Cut down the amount of time you spent on work or other activities	1	2
14. **Accomplished less** than you would like	1	2
15. Were limited in the **kind** of work or other activities	1	2
16. Had **difficulty** performing the work or other activities (for example, it took extra effort)	1	2

During the **past 4 weeks,** have you had any of the following problems with your work or other regular daily activities **as a result of any emotional problems** (such as feeling depressed or anxious)?

(Circle One Number on Each Line)

	Yes	No
17. Cut down the **amount of time** you spent on work or other activities	1	2
18. **Accomplished less** than you would like	1	2
19. Didn't do work or other activities as **carefully** as usual	1	2

20. During the **past 4 weeks,** to what extent has your physical health or emotional problems interfered with your normal social activities with family, friends, neighbors, or groups?

(Circle One Number)

Not at all 1

Slightly 2

Moderately 3

Quite a bit 4

Extremely 5

21. How much **bodily** pain have you had during the **past 4 weeks**?

(Circle One Number)

None 1

Very mild 2

Mild 3

Moderate 4

Severe 5

Very severe 6

22. During the **past 4 weeks,** how much did **pain** interfere with your normal work (including both work outside the home and housework)?

(Circle One Number)

Not at all 1

A little bit 2

Moderately 3

Quite a bit 4

Extremely 5

These questions are about how you feel and how things have been with you **during the past 4 weeks**. For each question, please give the one answer that comes closest to the way you have been feeling.

How much of the time during the **past 4 weeks** . . .

(Circle One Number on Each Line)

	All of the Time	Most of the Time	A Good Bit of the Time	Some of the Time	A Little of the Time	None of the Time
23. Did you feel full of pep?	1	2	3	4	5	6
24. Have you been a very nervous person?	1	2	3	4	5	6
25. Have you felt so down in the dumps that nothing could cheer you up?	1	2	3	4	5	6
26. Have you felt calm and peaceful?	1	2	3	4	5	6
27. Did you have a lot of energy?	1	2	3	4	5	6
28. Have you felt downhearted and blue?	1	2	3	4	5	6
29. Did you feel worn out?	1	2	3	4	5	6
30. Have you been a happy person?	1	2	3	4	5	6
31. Did you feel tired?	1	2	3	4	5	6

32. During the **past 4 weeks,** how much of the time has your **physical health or emotional problems** interfered with your social activities (like visiting with friends, relatives, etc.)?

(Circle One Number)

All of the time 1

Most of the time 2

Some of the time 3

A little of the time 4

None of the time 5

How TRUE or FALSE is each of the following statements for you.

(Circle One Number on Each Line)

	Definitely True	Mostly True	Don't Know	Mostly False	Definitely False
33. I seem to get sick a little easier than other people	1	2	3	4	5
34. I am as healthy as anybody I know	1	2	3	4	5
35. I expect my health to get worse	1	2	3	4	5
36. My health is excellent	1	2	3	4	5

SECTION D: QUALITY OF LIFE

Enter how satisfied you are with your situation in different aspects

		**Very Unhappy** 1	2	3	4	5	6	**Very Happy** 7
**D1.**	**Home and family**							
**D2.**	**Residence**							
**D3.**	**Working situation**							
**D4.**	**Economy**							
**D5.**	**Leisure time**							

As we pass through life, some changes may be felt in the course of years passing. Try to enter how you feel now.

		**Poor** 1	2	3	4	5	6	**Excellent** 7
**D6.**	**Hearing**							
**D7.**	**Vision**							
**D8.**	**Memory**							
**D9.**	**Physical fitness**							
**D10.**	**Appetite**							
**D11.**	**Temper**							
**D12.**	**Energy**							
**D13.**	**Patience**							
**D14.**	**Confidence(self esteem**							
**D15.**	**Sleep**							

		Not at all 1	2	3	4	5	6	Very much 7
**D16.**	**Do you feel important and appreciated outside your home?**							
**D17.**	**Do you feel important and appreciated in your home?**							

SECTION E: SOCIAL SUPPORT AND SOCIAL NETWORK

E1. How many people do you know and interact with, who have the same interests as you?

1. None
2. 1-2 people
3. 3-5 people
4. 6-10 people
5. 11-15 people
6. More than 15 people

E2. How many people that you know do you meet with, or speak to in an average week?
(Do not count people who you hardly will see again, for example customers in a shop)

1. None
2. 1-2 people
3. 3-5 people
4. 6-10 people
5. 11-15 people
6. More than 15 people

E3. Is that about enough people to meet, or would you like to meet more people in the average week?

1. Fewer
2. About enough
3. More

E4. How many friends do you have who can come to your house anytime and feel like being at home? (They wouldn't care if the house was a mess or if you were eating dinner. Family and relatives do not apply here)

1. None
2. 1-2 people
3. 3-5 people
4. 6-10 people
5. 11-15 people
6. More than 15 people

E5. How many people are there, in your family or amongst your friends, that you can talk openly to, without thinking carefully?

1. None
2. 1-2 people
3. 3-5 people
4. 6-10 people
5. 11-15 people
6. More than 15 people

E6. Is there a particular person who you feel you can really get support from?

1. No
2. Yes, but I don’t need it
3. Yes

E7. Is there a particular person who is especially close to you?

1. No
2. Not sure
3. Yes

E8. Do you have a particular person who you can share your innermost feelings with when you feel happy? Someone who is happy too, just because you are?

1. No
2. Yes

E9. Do you have a special person that you can share your innermost feelings with and confide in?

1. No
2. Yes

E10. Does someone occasionally hug you for comfort and support?

1. No
2. Yes

E11. Do you think you are truly appreciated at home or anywhere else?

1. Yes
2. Not enough
3. No

E12. Are there people around you who you easily can ask for things, for example people who you know well enough to borrow tools or kitchen equipment from?

1. No
2. Yes

E13. Besides the people at home, is there anyone you can turn to if you have problems? Someone you can easily meet and who you trust and who can really help you when you are in need of it?

1. No
2. Yes

E14. Have you during the past year participated in any leisure-time activity, voluntary organization etc. together with others (for example sports, study circle, theatre group, choir, political organization)?

1. Yes
2. No → Proceed to question F1

E15. How often do you participate in organizational activities in leisure time, club activities or study circles etc. with others?

1. 1-2 times per year
2. 1-2 times per month
3. 1-2 times per week
4. Every day
5. Don't know

E16. What kind of association/associations do you join?

1. Sports & exercise
2. Study circle
3. Theatre group
4. Choir
5. Other ………………………..

SECTION F: WORK

By "work" we mean your main occupation regardless if you are working in a place of business or at home. (Mark only the <u>one</u> alternative that's most suitable in your opinion)

		**Yes, often** 1	**Yes, some-times** 2	**No, seldom** 3	**No, hardly ever** 4
**F1.**	**Is your work physically demanding?**				
**F2.**	**Does your work demand that you are working fast?**				
**F3.**	**Is your work mentally demanding?**				
**F4.**	**Do you have enough time to finish your assignments?**				
**F5.**	**Are there conflicting demands in your work?**				
**F6.**	**Do you learn new things in your work?**				
**F7.**	**Does your work require skill?**				
**F8.**	**Does your work require ingenuity?**				
**F9.**	**Is your work repetitive?**				
**F10.**	**Do you have the freedom to decide how to preform your work?**				
**F11.**	**Do you have the freedom to decide what is to be preformed in your work?**				

F12. Do you usually have a chance to talk to your co-workers during breaks, if you would like to?

1. Yes, always
2. Yes, usually
3. No, I don’t have any breaks
4. No, I don’t have any breaks with co-workers

F13. Is your work of the character that you could leave it for a while to go talk to one of your co-workers?

1. Yes, mostly
2. Yes, sometimes
3. Only for urgent matters
4. No, it’s impossible

F14. Have you, as a part of your work, many contacts with your co-workers?

1. Yes, I have continuously many contacts
2. One or a couple of times per month
3. No, I mainly work alone
4. Seldom or never

F15. How often do you usually spend some time outside work together with a co-worker?

1. One or several times per week
2. One or a few times per month
3. One or a few times per year
4. Seldom or never

F16 When was the last time a co-worker came by to visit you at home?

1. One to four weeks ago
2. One to twelve months ago
3. More than a year ago
4. Never been visited by a co-worker

SECTION G: PHYSICAL ACTIVITY

G1. Mark in the chart below how you usually travel to and from your work each of the four seasons

(Only mark ONE square per season, please)

	**Spring**	**Summer**	**Fall**	**Winter**
1 Car				
2 Bus				
3 Walking				
4 Ride a bicycle				

How many kilometers do you have to travel to commute? (One way) ……………….

G2. Mark the alternative that best describes your work

1. Sitting still or standing
2. Light, but partially mobile
3. Light and mobile
4. Sometimes physically heavy
5. Physically heavy most of the time

G3 What recreational activities do you participate in, that requires some physical effort? (Mark suitable square for each line)

	**Never**	**1-2 times/month**	**3-4 times/month**	**2-3 times/week**	**Every day**
A. Walks					
B. Bicycling					

G6. How often have you worked out or exercised in your training-clothes during the last three months, with the purpose to improve your fitness or wellness?

1. Never
2. Now and then – not regularly
3. Once a week
4. 2-3 times/week
5. More than 3 times/week

G9. To what extent have you been physically active during leisure time during the past 12 months?
If this varies between seasons, try to state an average. Please mark ONE alternative only!

1. Sedentary leisure time *You are mainly spending your leisure time to reading, watching TV, cinema, or other sitting activities and you are walking, bicycling or moving in other way* *<u>less than 2 hours per week</u>*

2. Moderate physical activity during leisure time *You are walking, bicycling or moving in other way* *<u>at least 2 hours per week</u>* *usually* *<u>without sweating.</u>* *This includes for example walking or bicycling to and from your workplace, other walks, hard housework, ordinary gardening, go fishing, table tennis, bowling*

3. Regular moderate physical activity. *You are physically active on a regular basis* *<u>at least 30 minutes 1-2 times per week</u>* *with for example running, swimming, tennis, badminton or other activity that makes you sweat*

4. Regular physical activity and exercise. *You are running, swimming, playing tennis or badminton, or going to fitness-classes* *<u>at least 30 minutes</u>* *each time and on average* *<u>at least 3 times</u>* *a week.*

G10. During an ordinary week, how much time do you spend on moderately strenuous activities that makes you warm) for example swift walking, gardening, heavy house work, bicycling, swimming. This could vary during a year, but state an average. Only mark one alternative!

1. 5 hours per week or more
2. More than 3 but less than 5 hours per week

3. 1-3 hours per week
4. At most 1 hour per week
5. Not at all
6. I do not know/cannot take my stand

G11. During the last 7 days, how much time did you usually spend sitting while at work, doing course work, during transportation/commuting, at home and during leisure time. This may include time spent sitting at a desk, going by bus or car, visiting friends, to be sitting while eating or talking or reading, or sitting or lying down to watch a movie or television

A On a **weekday**? **hours …… minutes …… per day** **Do not know…….**

B On a **weekend**? **hours …… minutes …… per day** **Do not know…….**

SECTION G: SMOKING AND SNUFF

H1. Do you presently smoke? (Several alternatives may be marked)

1. No, I never have smoked → Proceed to question H4
2. Yes, I smoke cigarettes — Number of cigarettes / day
   - ❑ 1-4
   - ❑ 5-14
   - ❑ 15-24
   - ❑ 25 or more
3. Yes, I smoke cigars
4. Yes, I smoke a pipe
5. Yes, I smoke occasionally (Not daily) — How freqently do you smoke?
   - ❑ less than one during 1 day per month
   - ❑ 1-3 days per month
   - ❑ Usually one day per week
   - ❑ Usuallu during 2-4 days per week
   - ❑ AQlmost every day
6. Not now, but I used to smoke daily
7. Not now, but I used to smoke occasionally → Proceed to question H3

H2. How old were you when you started smoking? …………years old

H3. How old were you when you stopped smoking? …………years old

H4. Have you ever used snuff? (only mark one alternative!)

1. No → If you never have smoked, proceed to question H7
   → If you smoked previously, proceed to question H6B
2. Yes, I used to, but I don't anymore
3. Yes, I do. Less than 2 boxes / week
4. Yes, I do. 2-4 boxes / week
5. Yes, I do. More than 4 boxes but less than 7 boxes / week
6. Yes, I do. More than 7 boxes / week

H5. For how long time have you been using snuff? …… years

H6. Did you start using snuff to quit smoking?

1. Yes
2. No
3. I both smoke and use snuff

H6A. Did you use nicotine replacement therapy to quit using snus?
1. Yes
2. No

H6B. Did you use nicotine replacement therapy to quit smoking?
1. Yes
2. No

H6C. Do you still use medication against nicotine dependence in spite of neither smoking nor using snus?
1. Yes
2. No

SECTION I: SLEEPING HABITS

H7. What risk do you run to doze off or fall asleep in the following situations, instead of just being tired? This relates to your usual way of living during the last weeks. If you did not experience all these situation recently, try to evaluate how each situation would have influenced you?

		**No risk** 1	**Small risk** 2	**Moderate risk** 3	**High risk** 4
**a**	**Sitting reading**				
**b**	**Watching TV**				
**c**	**Sitting passive on an public place (for example theater, on a meeting)**				
**d**	**Passenger in a car one hour without break**				
**e**	**Lying down for a little rest in the afternoon if the circumstances allow it**				
**f.**	**Sitting and talking to somebody**				
**g**	**Is sitting still after lunch (without any alcohol)**				
**h**	**In a car that has stopped a few minutes in the traffic**				

H8. Snoring and apnoes

		Yes always 1	Yes, almost always 2	Yes Sometimes 3	No Almost never 4	No Never 5	I don't know 6
**a**	**Do you snore while sleeping?**						
**b**	**Did your wife/husband/spouse notice that you have apnoeas (respiratory arrests) while sleeping?**						

SECTION J: ALCOHOL HABITS

One glass means:
50 cl “Folk” beer, 2.25 - 3.5 % Alcohol by volume
33 cl Strong beer, >3.5 % Alcohol by volume
One glass of red or white wine
A small glass of dessert wine/wine with a high alcohol content
4 cl sprits for example whiskey

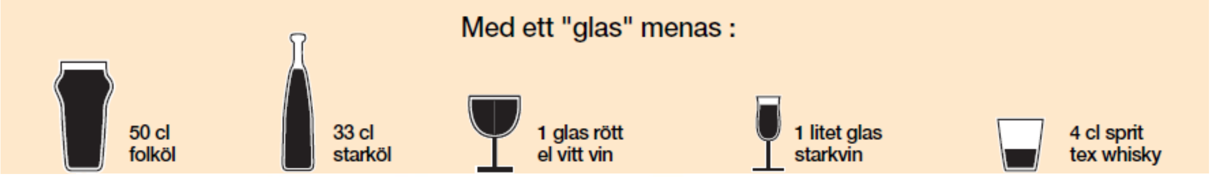

Place an X in one box that best describes your answer to each question.

Questions	0	1	2	3	4	
1. How often do you have a drink containing alcohol?	Never	Monthly or less	2 to 4 times a month	2 to 3 times a week	4 or more times a week	
2. How many drinks containing alcohol do you have on a typical day when you are drinking?	1 or 2	3 or 4	5 or 6	7 to 9	10 or more	
3. How often do you have five or more drinks on one occasion?	Never	Less than monthly	Monthly	Weekly	Daily or almost daily	
4. How often during the last year have you found that you were not able to stop drinking once you had started?	Never	Less than monthly	Monthly	Weekly	Daily or almost daily	
5. How often during the last year have you failed to do what was normally expected of you because of drinking?	Never	Less than monthly	Monthly	Weekly	Daily or almost daily	
6. How often during the last year have you needed a first drink in the morning to get yourself going after a heavy drinking session?	Never	Less than monthly	Monthly	Weekly	Daily or almost daily	
7. How often during the last year have you had a feeling of guilt or remorse after drinking?	Never	Less than monthly	Monthly	Weekly	Daily or almost daily	
8. How often during the last year have you been unable to remember what happened the night before because of your drinking?	Never	Less than monthly	Monthly	Weekly	Daily or almost daily	
9. Have you or someone else been injured because of your drinking?	No		Yes, but not in the last year		Yes, during the last year	
10. Has a relative, friend, doctor, or other health care worker been concerned about your drinking or suggested you cut down?	No		Yes, but not in the last year		Yes, during the last year	

J11. Have other people annoyed you by criticizing your drinking?

1. Yes
2. No

J12. Have you ever felt you needed to cut down on your drinking?

1. Yes
2. No

SECTION L: EATING HABITS

These are questions about your eating-habits in the last year. Mark the choice that most accurate for you.

L1. Which of the following alternatives for breakfast is the best match for your own.

0. Only coffee / tea
1. Coffee / tea and a sandwich
2. Coffee / tea and wheat-bread or biscuits
3. Cereal with or without sandwich
4. Oatmeal with or without sandwich
5. Gruel with or without sandwich
6. I don't eat breakfast

L2. Have you taken any of the following dietary supplements?

During the last year

1. No dietary supplements
2. Multi vitamins
3. Multi minerals
4. Iron
5. Selenium
6. Other

During the last 14 days

1. No dietary supplements
2. Multi vitamins
3. Multi minerals
4. Iron
5. Selenium
6. Other

L3. Which of the following alternatives is the most applicable for you? (only mark one alternative)

1. I have a normal diet (meat, fish etc.)
2. I have a vegan diet (no food that's based on animals)
3. I am a lacto-vegetarian (with milk-cheese)

L4. Look at the pictures below and mark for each item the letter that most looks like your average portion size.

Potato/rice/macaroni	A	B	C	D
Meat/fish	A	B	C	D
Vegetables	A	B	C	D

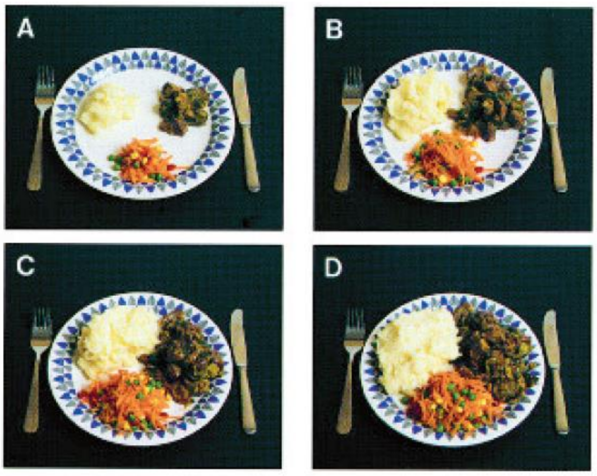
A
B
C
D

SECTION L: FOOD FREQUENCY QUESTIONNAIRE

How often do you eat the foods below?
Mark your average consumption during the last year. (Only one alternative per row)

		Never 0	Few times per year 1	1-3 times per month 2	Once a week 3	2-3 times per week 4	4-6 times per week 5	Once a day 6	2-3/ times per day 7	4 or more times per day 8
01.	“Bregott” on a sandwich									
02.	Butter on a sandwich									
03.	Low fat margarine on a sandwich									
04.	Margarine on a sandwich									
05.	Butter for cooking									
06.	Margarine for cooking									
07.	Oil for cooking									
08.	Salad dressing with oil									
09.	Cream, cream fraiche,sour cream									
10.	Hard bread (e.g. “Husmans”)									
11.	Whole meal bread									
12.	White bread									
13.	Biscuits, buns									
14.	Medium-fat Cheese (28% fat)									
15.	Low-fat cheese (10-17% fat)									
16.	Sausage or pâté on sandwich									
17.	Meat on sandwich									
18.	Porridge (Oatmeal, whole wheat, rye, or barley)									
19.	Rosehip.-or other fruit-soup									
20.	Yogurt, sour milk									
21.	Light yogurt, light sour milk									
22.	Muesli (flake & grain)									
23.	Corn-flakes, K-special etc									
24.	Berries, fresh or frozen									
25.	Apples, pears, peaches, oranges, tangerine, grape fruit									
26.	Bananas									
27.	Carrots, root-crops									
28.	Tomato, cucumber									
29.	Salad, spinach, broccoli, cabbage									
30.	Cooked or baked potatoes									
31.	Fried potato, french-fries									
32.	Rice									
33.	Spaghetti, macaroni									
34.	Beans, pea-soup, other beans									
35.	Pancakes, waffles, potato dumplings									
36.	Pizza									
37.	Dishes based on Minced meat									

38.	Meat stews									
39.	Whole meat (steaks, chops)									
40.	Bacon									
41.	Sausage as a dish									
42.	Hamburgers									
43.	Chicken, poultry									
44.	Egg									
45.	Low-Fat fish (bass, codfish)									
46.	High fat fish (herring, salmon)									
47.	Salted fish (salted herring)									
48.	Smoked fish, smoked meat									
49.	Ice-cream									
50.	Sweets (chocolates, candy)									
51.	Sugar, honey, marmalade, jam									
52.	Cookies, pastries									
53.	Chips, popcorn, salted peanuts etc.									
54.	Low-fat milk (0.5% fat)									
55.	Medium milk (1.5% fat)									
56.	Standard milk (3% fat) “home milk”									
57.	Soft drinks, Coca-Cola, lemonade, nectar, juice									
58.	Regular coffee (filtered coffee)									
59.	“Boiled” coffee									
60.	Tea									
61.	Light beer < 2.25 % Alcohol by volume									
62.	“Folk” beer 2.25 - 3.5 % Alcohol by volume									
63.	Strong beer >3.5 % Alcohol by volume									
64.	Wine									
65.	Hard liquor, spirits									
66.	Water									

Translation July 2000 by Simon Weinehall
